# Influence of Familial Renal Glycosuria Due to Mutations in the *SLC5A2* Gene on Changes in Glucose Tolerance over Time

**DOI:** 10.1371/journal.pone.0146114

**Published:** 2016-01-06

**Authors:** Emilia Ottosson-Laakso, Tiinamaija Tuomi, Björn Forsén, Monika Gullström, Per-Henrik Groop, Leif Groop, Petter Vikman

**Affiliations:** 1 Diabetes and Endocrinology, Department of Clinical Sciences, Lund University, Malmö, Sweden; 2 Folkhälsan Institute of Genetics, Folkhälsan Research Centre, Biomedicum, Helsinki, Finland; 3 Research Program Unit, Diabetes and Obesity, University of Helsinki, Helsinki, Finland; 4 Närpes Health Care Center, Närpes, Finland; 5 Abdominal Center Nephrology, University of Helsinki and Helsinki University Hospital, Helsinki, Finland; 6 Baker IDI Heart and Diabetes Institute, Melbourne, Australia; 7 Finnish Institute of Molecular Medicine, University of Helsinki, Helsinki, Finland; Emory University, UNITED STATES

## Abstract

Familial renal glycosuria is an inherited disorder resulting in glucose excretion in the urine despite normal blood glucose concentrations. It is most commonly due to mutations in the *SLC5A2* gene coding for the glucose transporter SGLT2 in the proximal tubule. Several drugs have been introduced as means to lower glucose in patients with type 2 diabetes targeting SGLT2 resulting in renal glycosuria, but no studies have addressed the potential effects of decreased renal glucose reabsorption and chronic glycosuria on the prevention of glucose intolerance. Here we present data on a large pedigree with renal glycosuria due to two mutations (c.300-303+2del and p.A343V) in the *SLC5A2* gene. The mutations, which *in vitro* affected glucose transport in a cell line model, and the ensuing glycosuria were not associated with better glycemic control during a follow-up period of more than 10 years. One individual, who was compound heterozygous for mutations in the *SLC5A2* gene suffered from severe urogenital candida infections and postprandial hypoglycemia. In conclusion, in this family with familial glycosuria we did not find any evidence that chronic loss of glucose in the urine would protect from deterioration of the glucose tolerance over time.

## Introduction

Familial renal glycosuria (FRG) is a rare disorder that is characterized by decreased renal reabsorption of glucose. In most published cases this abnormality is due to mutations in the *SLC5A2* gene, which encodes for the sodium glucose co-transporter 2, SGLT2 [1–6]. SGLT2 is the major glucose co-transporter in the proximal tubule responsible for 90% of the renal glucose reabsorption while 10% is absorbed by the more distally located sodium glucose co-transporter 1 (SGLT1). It is noteworthy that SGLT2 also is the target for antidiabetic therapy. FRG is usually inherited in a co-dominant fashion with incomplete penetrance [2]. Although glucose loss in the urine can range from 1 to 150 g/1.73m^2^ FRG is generally considered as a benign condition, apart from anecdotal reports of polyuria, increased frequency of urinary tract infections and activation of the renin-angiotensin aldosterone system [7–9]. No studies have addressed the potential effects of decreased renal glucose reabsorption and chronic glycosuria on the prevention of glucose intolerance.

Early studies in rats reported beneficial effects of inhibition of the renal glucose reabsorption by phlorizin on the glucose tolerance and prompted the introduction of SGLT2 inhibitors as means to lower glucose in patients with type 2 diabetes. This treatment strategy was further justified by findings of increased SGLT2 expression and increased glucose uptake in the proximal tubule in biopsies from type 2 diabetes patients [10] although it is not known whether the increased expression is a cause or consequence of hyperglycemia. Furthermore, it has been shown that inhibition of glucose reabsorption by SGLT2 inhibitors increases endogenous glucose production and plasma glucagon concentrations in diabetic subjects [11,12], and nondiabetic mice [13]. Notably, SGLT2 is also expressed in the alpha cells alongside SGTL1 [14]. Both of these glucose transporters are down regulated in islets from donors with type 2 diabetes and in *ob/ob* mice after the development of hyperglycemia together with an increased expression of glucagon mRNA [14].

Several SGLT2 inhibitors have been introduced for the treatment of type 2 diabetes and are also being tested in patients with type 1 diabetes. The therapeutic window of this class of drugs has been considered good with beneficial effects on weight and blood pressure and few other side effects than a slightly increased frequency of genital mycotic infections that seldom leads to discontinuation of the drug [15,16]. Therefore, these new drugs could also be considered for the prevention of diabetes. However, studies on the potential effects of SGLT2 inhibitors on prevention of diabetes are lacking. One possibility to address this question without testing the drug in a non-diabetic population would be to study non-diabetic carriers with FRG for their propensity to develop diabetes or deterioration of glucose tolerance. We did this in a large pedigree with FRG followed by repeated oral glucose tolerance tests for more than 30 years, after first confirming that their FRG was due to mutations in the *SLC5A2* gene. In this family we found no evidence that chronic loss of glucose in the urine would protect from deterioration of the glucose tolerance over time.

## Materials and Methods

### Subjects

**Fig 1:**
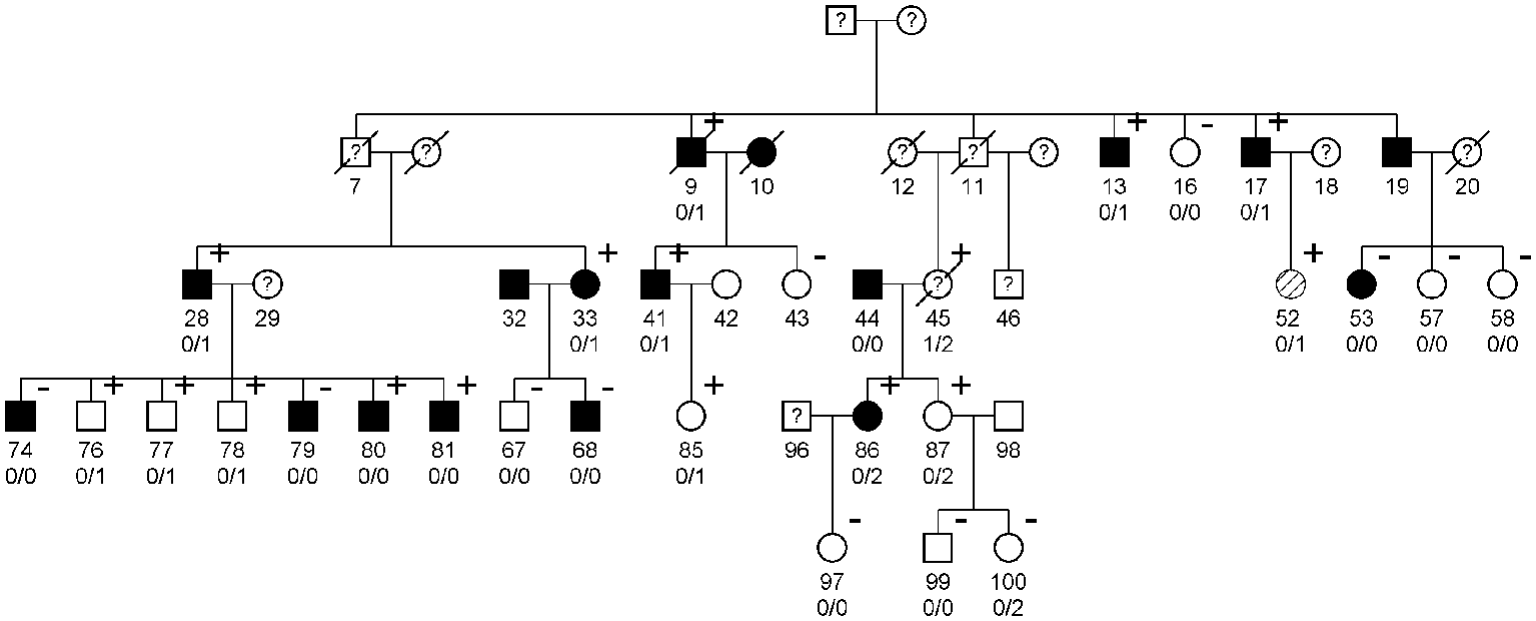
The pedigree of the family with glycosuria. Black symbols indicate that the person has IFG, IGT or T2D (squares = men, circles = females), white symbols indicate normal glucose tolerance. Diagonally hatched symbol indicates T1D (not included in the analysis). Plus and minus indicates positive or negative test for glycosuria. First number under symbols indicates ID number. Genotyping of the deletion (c300-303+2del) showed that 10 of 22 genotyped family members are heterozygous for the mutation. The carriers of the deletion (c300-303+2del) are denoted with 0/1 and carriers of the reference allele are denoted with 0/0 while carriers of the missense mutation (p.A343V) are denoted with 0/2.

A family with renal glycosuria from the Botnia region on the west coast of Finland has been followed clinically for the last 30 years [17]. In addition to renal glycosuria, 8 individuals developed impaired fasting glucose (IFG), impaired glucose tolerance (IGT) or type 2 diabetes (T2D) (Fig 1) and the family were therefore followed by repeated oral glucose tolerance tests (OGTT, 75 g glucose) as part of the Botnia Study [18]. To evaluate whether glucose loss in the urine in individuals with glycosuria would affect glucose tolerance we calculated the changes in the area under the OGTT curve (ΔAUC_OGTT_) between the first and last visit (mean follow up time 10.5 years, range 3–22 years). AUC_OGTT_ was calculated with the trapezoidal method from the plasma glucose concentrations at 0, 30, 60 and 120 minutes during the OGTT. One female family member (No 45 in the pedigree, Fig 1) presented with marked glycosuria associated with symptoms of postprandial hypoglycemia and severe chronic urinary tract infections (predominantly candida infections). Under controlled conditions with a daily intake of 160 grams of carbohydrates, she excreted 55.2 g glucose/24 hours. She died from a gastric cancer at the age of 49 years. A paraffin embedded gastric biopsy was used for extraction of her DNA. Thin sections of the tissue were carefully scraped off the block using a scalpel and put into xylene for paraffin removal and the DNA was extracted using the QIAmp DNA FFPE Tissue Kit (Qiagen, Hilden, Germany) according to the manufacturer’s instructions.

The DNA extracted from the paraffin embedded tissue was unfortunately not of sufficient quality for sequencing; therefore two potentially functional mutations (c.300-303+2del and p.A343V) were imputed from sequencing data from her husband, children and grandchildren and then genotyped in the index case, family member 45, using a Taqman assay and a PCR with genotype specific primers (S2 Table).

To investigate the prevalence of glycosuria in the general population we extracted data on urine glucose excretions during OGTT from 4999 individuals part of the Botnia Study [18]. This was related to diagnosis of IGT/T2D using the Χ^2^ test.

The study was approved by the ethics committee at Vaasa Central Hospital, and written informed consent was obtained from all participants or guardians on the behalf of the minors/children participants.

### Whole exome sequencing and validation genotyping

DNA was extracted from blood samples and one μg of high quality DNA was used for sample preparation for sequencing using the TruSeq DNA sample preparation kit v2 (Illumina, CA, USA) before whole exome capture with the TruSeq Exome Enrichment Kit (Illumina, CA, USA) according to the manufacturer’s instructions. A paired end 2x100 base pair sequencing was then performed on a HiSeq 2000 (Illumina, CA, USA).

After base calling and de-multiplexing using CASAVA 1.8.2 (Illumina, CA, USA) the fastq-files were quality assessed with FastQC (http://www.bioinformatics.babraham.ac.uk/projects/fastqc/) and aligned against Hs37d5 using Burrows-Wheeler Aligner (BWA) v. 0.6.8 [19]. The Genome Analysis Tool Kit [20] (GATK) v. 1.6.2 was used on the resulting BAM-files for local realignment around known indels (IndelRealigner), for marking of duplicate reads (MarkDuplicates) and for base quality recalibration (CountCovariates and TableRecalibration). Variants were called using GATKs Unified Genotyper and variant recalibration (VariantRecalibrator and ApplyRecalibrations) [21]. This procedure was done according to the GATK best practices (https://www.broadinstitute.org/gatk/guide/best-practices).

As most mutations that cause FRG are located in the *SLC5A2* gene [22], the initial filtering was restricted to variants within the region of this gene. Resulting variants were analysed with SnpEff v3.0 [23] and SIFT [24] to evaluate functionality and potential effect of the variants on the resulting protein. Only variants predicted to be detrimental to the protein were tested for association with renal glycosuria in the family using Fisher’s exact test. The ExPASy Translate tool [25] was used to predict the effect of mutations on the translation of the mRNA.

The selected variants were validated in 23 family members by genotyping using custom made TaqMan assays (Life Technologies, CA, USA) according to the supplier’s instructions.

### Functional studies expressing SGLT2 mutation in HEK293-cells

We also tested whether the missense mutation in the *SLC5A2* gene (see below) influenced glucose transport and reabsorption in the tubule by expressing the wild type (WT) and mutant form of the protein (SGLT2) in HEK 293 [26] cells lacking the natural SGLT2 [27]. The WT cDNA sequence (S1 table) of *SLC5A2* was synthesized and mutated (GenScript USA Inc., NJ, USA) and the resulting cDNA was cloned into the neomycin resistance containing pCMV6-Neo vector (OriGene Technologies, Inc., MD, USA). The constructs were separately transfected into the HEK293 cell line in triplicates, and successfully transfected cells were selected using 0.8 mg/ml of G418 (Merk Millipore, MA, USA), an antibiotic often used to select for neomycin resistant cells, for 14 days. The cells were subsequently cultured in D-MEM (Cat# 41966029, Life Technologies, CA, USA) supplemented with 10% fetal bovine serum and 1% penicillin/streptomycin with a 50 μg/ml maintenance dose of G418. *SLC5A2* mRNA expression was measured by SYBR-green qPCR using *SLC5A2* mRNA specific primers designed to span exon junctions (S2 Table).

To evaluate the effect of the missense mutation in *SLC5A2* on glucose transport the transfected HEK293 cells were assayed using the Glucose Uptake Cell based Assay Kit (Cayman Chemical, MI, USA). Triplicates of the WT and mutant SGLT2 cell lines and the untransfected HEK 293 cell line were seeded at 2x10^4^ cells per well in a 96-well plate and incubated over night. The relative mRNA expression was measured to compare the transfection and transcription efficiency of the six cell lines. The next morning, the cells were incubated in glucose and serum free medium containing 150 μg/ml of the fluorescent glucose analogue 2-deoxy-2[(7-nitro-2,1,3-benzoxadiazol-4-yl)amino]-D-Glucose (2-NBDG) for 30 minutes. The cells were then washed and treated according to the manufacturer’s instructions before measuring fluorescence of the cells using an Infinite 200 Pro plate reader (Tecan, Männedorf, Switzerland). Untransfected cells were treated with the glucose uptake inhibitor Apigenin to control for unspecific uptake and the signal was used as background in the measurements. Relative glucose uptake was calculated as fluorescence normalized against WT-transfected signal and the difference between the WT-transfected cells and the mutant-transfected cells was tested with Student’s t-test.

### Screening for SGLT2 mutations in the Botnia Study

To study how common these mutations in the *SLC5A2* gene are in the general population we screened 2584 samples from the Botnia study [18] for the c.300-303+2del mutation by genotyping them using a custom TaqMan assay (Life Technologies, CA, USA). Secondly, we screened whole exome sequencing data from the GOT2D consortium for possibly damaging variants in a total of 2760 samples.

### *SLC5A2* and *SLC5A1* expression in human pancreatic islets

As the expression of both *SLC5A2* and *SLC5A1* has been suggested to be affected in islets from diabetic patients [14] we studied the expression of these two genes in 132 human islet samples from donors with a wide range of glycemia [28] using STAR[29]-aligned RNA-sequence data by the voom [30] and limma [31] R-packages. Expression values were expressed as the logarithmic count per million (log2CPM). The expression of *SLC5A2* and *SLC5A1* was correlated with insulin and glucagon expression levels and *SLC5A2* expression with glucagon secretion at 1 mM glucose using Pearson correlation. All p-values were FDR corrected. The islets were provided by the Human Tissue Lab EXODIAB/LUDC through the Nordic Transplantation Program (http://www.nordicislets.org).

## Results

### Mutations in the *SLC5A2* gene were associated with renal glycosuria in the family

By sequencing the *SLC5A2* gene in 23 members of the FRG family three novel and one previously reported variant were found, i.e. 3 single nucleotide variants (SNVs) and one 6 base pair deletion (Table 1). Two SNVs were intronic in no known regulatory region, and the third SNV was only seen in one of 23 samples, and therefor they were not considered any further. The deletion (c.300-303+2del) removed the four last base pairs in exon three as well as two base pairs of the conjunctive splice site and was by the ExPASy Translate tool [25] predicted to cause a premature stop codon, resulting in a shorter 117 residue peptide (S1 Fig).

**Table 1:** Variants found in the *SLC5A2* gene by whole exome sequencing of the family.

Chromosome:position	ID	REF/ALT	Intron/exon	SNPEff prediction	SIFT prediction
16: 31495577*	rs9924771	A/G	Intron		
16: 31495671*	rs11646054	G/C	Intron		
16: 31495873	rs9934336	G/A	Intron		
16: 31496240	c.300-303+2del	GGAATGT/G	Exon	FRAME_SHIFT(HIGH), SPLICE_SITE_DONOR(HIGH)	N/A^†^
16: 31496471	.	A/C	Intron		
16: 31498364*	rs5816530	T/TCAAAAA	Intron		
16: 31499713	p.A343V	C/T	Exon	NON_SYNONYMOUS_CODING(MODERATE)	Damaging

^†^ Only SNVs are scored by SIFT. The variants found in the region of *SLC5A2* in the whole exome sequencing and the predicted functional consequence of the variants, where applicable. The possible predictions of SNPEff are High, Moderate, Low and Modification, and by SIFT Tolerated or Damaging.

Variants marked with star (*) were found in the sequencing of the children and grandchildren of subject no 45.

**Fig 2:**
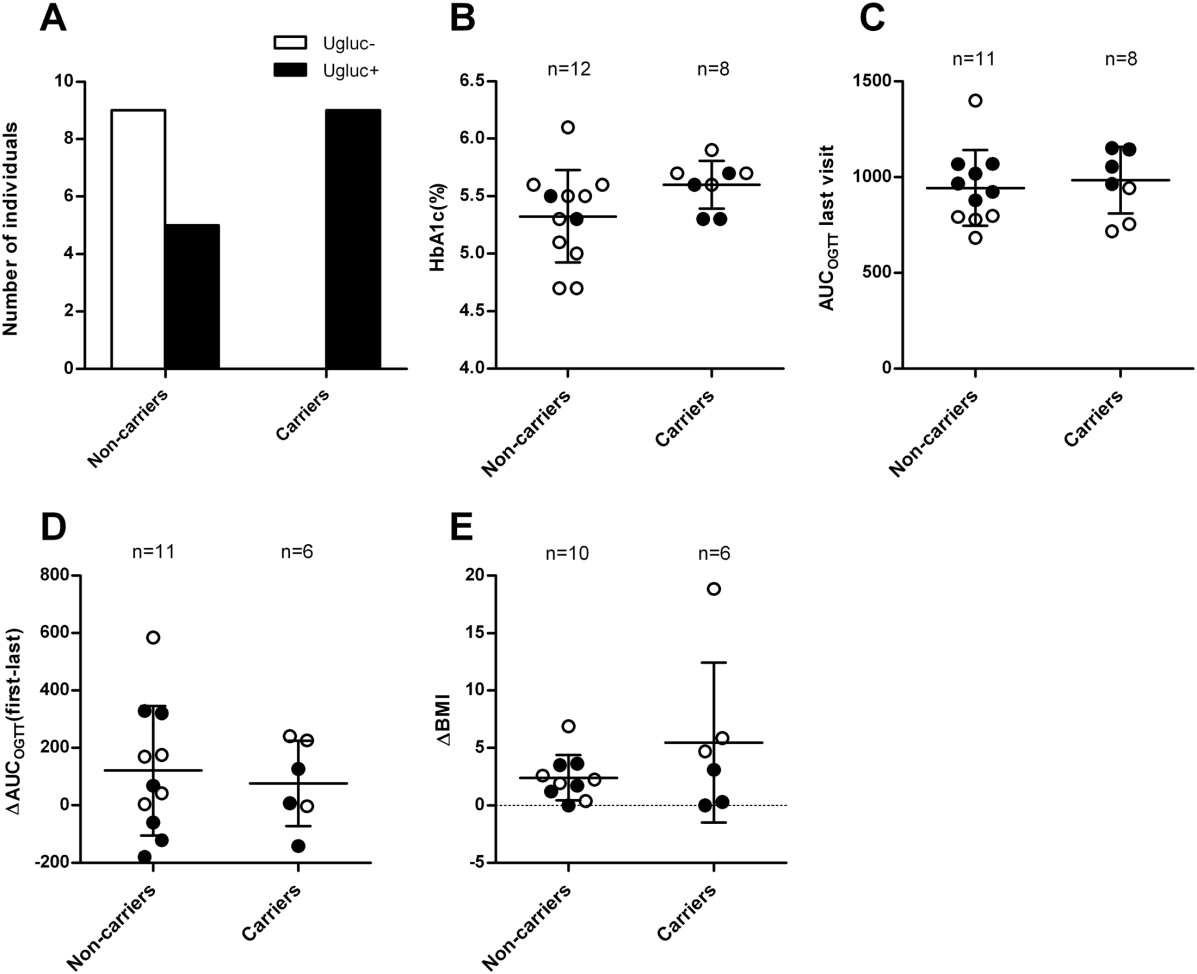
Carriers of the deletion are not protected against deterioration of glycemic status A) glycosuria in carriers and non-carriers of the mutation deletion (c300-303+2del). All but one of the non-carriers displaying glycosuria had impaired fasting glucose, impaired glucose tolerance or type 2 diabetes, B) HbA_1c_ at examination did not differ between carriers and non-carriers (p = 0.09), C) Area under the glucose curve during the last OGTT (p = 0.64), D) Change in area under the glucose tolerance from the first to the last OGTT (p = 0.67, mean follow up time 11 years), E) Change in BMI during the follow-up period. The white dots in the graphs represent non-diabetic individuals and the black dots individuals with impaired fasting glucose/impaired glucose tolerance/type 2 diabetes. The error bars show the SD.

This heterozygous deletion (c.300-303+2del) segregated with glycosuria in 10 of 14 family members (Fig 1). All carriers of the deletion (c.300-303+2del) excreted glucose in urine (defined as having at least one positive quantitative or qualitative urine glucose tests) compared with 5 out of 14 non-carriers (p = 0.003, Fig 2A). While this segregation was compatible with co-dominant inheritance, the index case (family member 45) with the most severe form of glycosuria, excreting 55.2 g glucose/24 hours compared to other family members excreting between 0.2–20.7 g/24 hours, had only one copy of the mutation and, in contrast to our expectations, was not homozygous for the deletion (see below).

In order to impute the genotype of the index case we genotyped her husband, her two children and three grandchildren. Her husband did not carry any mutation in the *SLC5A2* gene but 4 variants were found in the children and the grandchildren (Table 1). Three of the variants were intronic whereas the fourth variant (p.A343V) was a missense mutation in exon 9 of the *SLC5A2* gene also found in its heterozygous form in the daughters and one grandchild. Both daughters presented with glycosuria while the grandchild did not (Table 2).

**Table 2:** The p.A343V mutation in the family of the index case (No 45).

ID	Mutation	Urine glucose
86	Yes	+
87	Yes	+
97	No	-
99	No	-
100	Yes	-

As the SNV was not found in the father of the offspring, it is likely that the missense mutation (p.A343V) is inherited from the index case suggesting that she would be compound heterozygous for the deletion (c.300-303+2del) and the missense mutation (p.A343V). To test this we performed TaqMan genotyping of the 6 bp deletion and PCR using genotype specific primers for the missense mutation of DNA from the index case. These analyses confirmed our hypothesis that she indeed was compound heterozygous for mutations in the *SLC5A2* gene as she carried both the 6 bp deletion (c.300-303+2del) and the missense mutation (p.A343V, S2 Fig).

### Glucose transport in kidney cells (HEK293) is impaired in carriers of the p.A343V mutation

**Fig 3:**
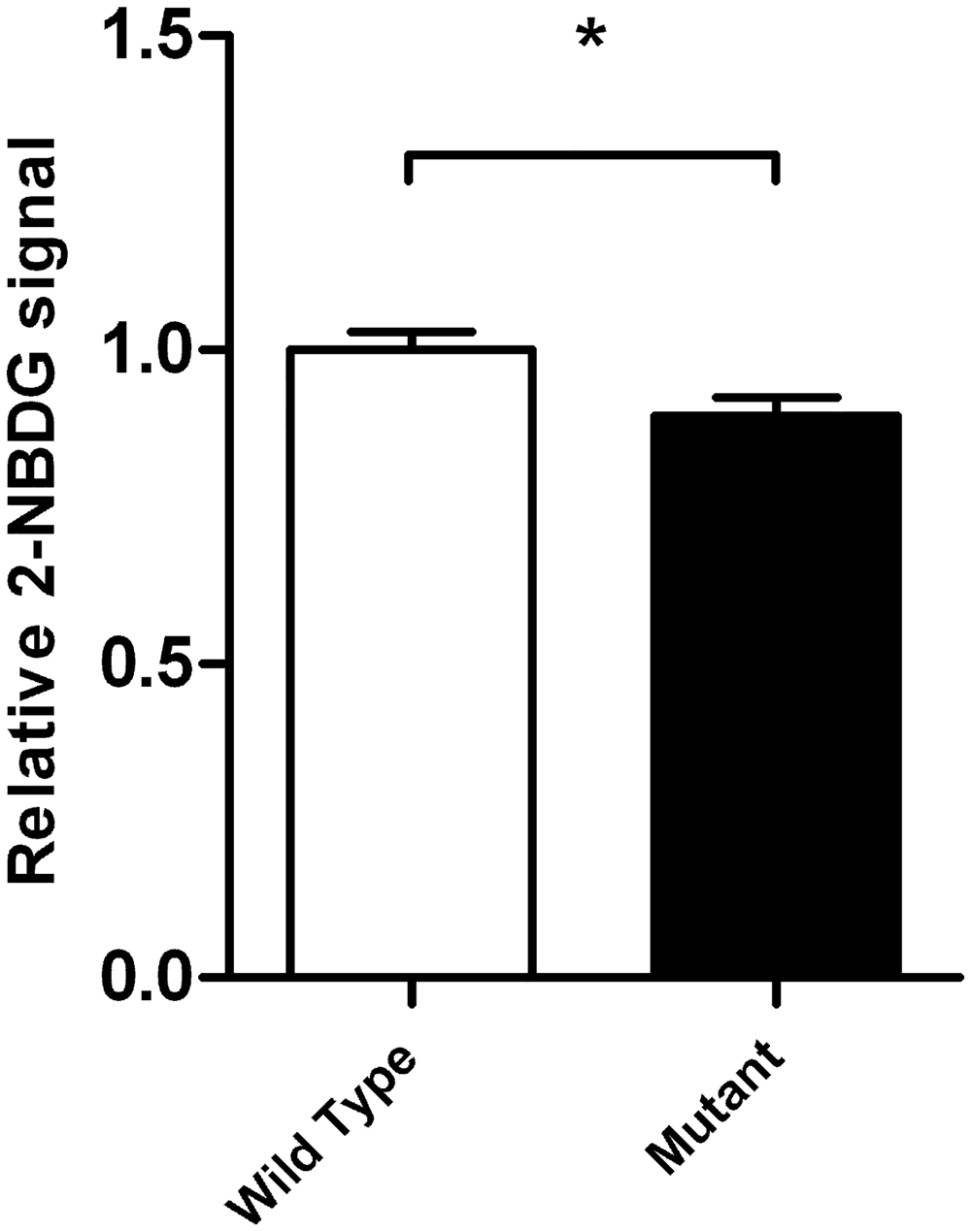
The p.A343V mutated SGLT2 protein transports less glucose across the cell membrane. Glucose uptake of the mutant HEK293 cells was lower as compared to wild type SGLT2 (p = 0.02). The open bar describes the relative fluorescent signal as measured and calculated by the glucose uptake assay in the HEK293 cell lines transfected with wild type *SLC5A2* (n = 12). The closed bar describes the HEK293 cell lines transfected with p.A343V mutated *SLC5A2* (n = 12). The difference was tested between wild type-transfected and mutant-transfected cell lines using student’s t-test. The error bars show the SEM, *p<0.05.

To confirm that the missense p.A343V mutation affected the glucose transport we expressed the mutation in a human kidney cell line, HEK293, lacking the endogenous SGLT2 protein. The cells expressing the p.A343V transporter showed decreased capability to transport glucose over the cell membrane *in vitro* relative to the cells expressing the WT transporter (n = 12, p = 0.02, Fig 3).

### Influence of the *SLC5A2* mutations on change in the glucose tolerance over time

Of 13 glycosuric family members, 5 had normal whereas 8 had abnormal glucose tolerance. To confirm that the glycosuria was due to a reduced threshold for glucose reabsorption in the kidney in these family members and not only a corollary of elevated plasma glucose we compared the prevalence of glycosuria between individuals with normal and abnormal glucose tolerance (IGT or T2D) and between those with the *SLC5A2* mutations (6 of whom also had abnormal glucose tolerance). Among 4999 individuals from the Botnia study, 95% of all glycosuric individuals had IGT or T2D and, vice versa, 64% of patients with IGT/T2D had glycosuria (Χ^2^ p<0.0001). Although glycosuria was also present in 9.5% of the individuals with normal glucose tolerance, these individuals had significantly higher glucose area under the curve during OGTT compared to those without glycosuria (n = 323, 1005 mmol·l^-1^·min compared to 812 mmol·l^-1^·min, p = 0.001). To explore how common the c.300-303+2del mutation was in Finland we genotyped 2584 individuals from Botnia for the c.300-303+2del mutation and identified 10 additional individuals from 5 families with the deletion. Next we analysed whole exome sequence data from the GOT2D consortium including individuals from the Botnia region. Of the 2760 screened individuals two carried the c.300-303+2del mutation and they both came from the Botnia region. Both were glycosuric but they had also diabetes. In addition, in the GOT2D data set we found 36 other missense variants in the *SLC5A2* gene, 7 of which were previously reported in dbSNP [32].

To test whether chronic glycosuria as seen with the use of SGLT2 inhibitors would prevent diabetes or deterioration of the glucose tolerance we assessed changes in the glucose tolerance over time in glycosuric carriers and non-carriers of the mutation. As seen in Fig 2, there was no protective effect of the loss of glucose in the urine with respect to change in glucose tolerance (p = 0.67, Fig 2D, S3–S4 Figs) or HbA_1c_ (p = 0.09 Fig 2B) over time in the mutation carriers. There was also no difference in glucose tolerance at the last visit (p = 0.64, Fig 2C). Since treatment with SGLT2 inhibitors is associated with decreased body weight, an increase in the haematocrit values [33] and an increase in total, LDL and HDL cholesterol concentration [34] we also examined changes in BMI, hematocrit and cholesterol between carriers and non-carriers. We did not observe any differences in the change in BMI (p = 0.33, Fig 2E and S5 Fig), cholesterol (p = 0.14) or haematocrit (p = 0.10) between the two groups. There was furthermore no difference in the change in triglycerides (p = 0.88) or HDL cholesterol (p = 0.20) between the groups.

### *SLC5A1*, but not *SLC5A2* expression correlates with glucagon in human pancreatic islets

**Fig 4:**
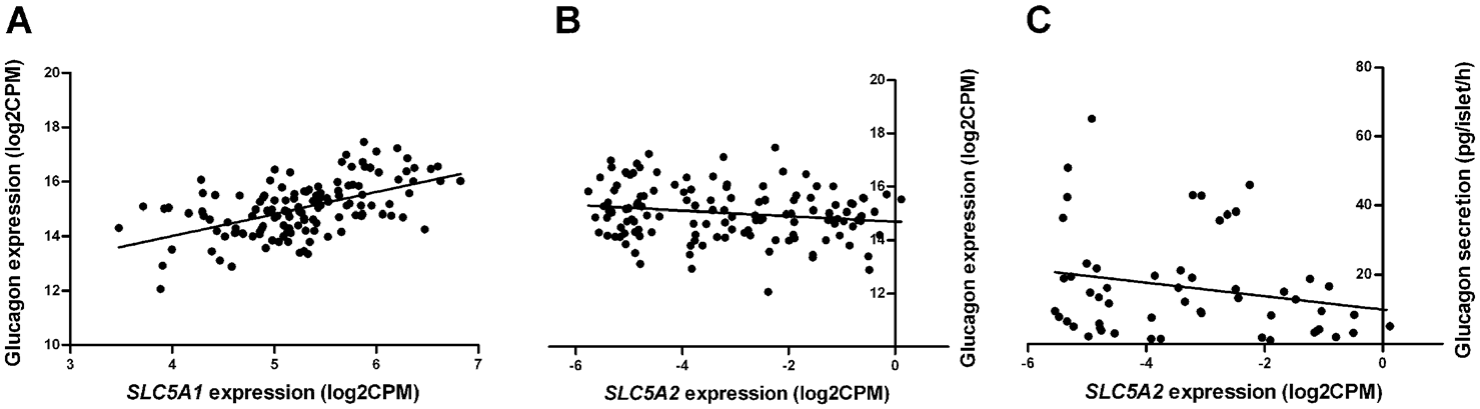
The gene expression of *SLC5A2* and *SLC5A1* in human pancreatic islets correlated with glucagon gene expression. The mRNA expression of *SLC5A1* (panel A, n = 131, r = 0.53, q-value<0.0001), but not *SLC5A2* (panel B, n = 131, r = -0.15, q-value = 0.19), correlates with the expression of the glucagon gene or secretion of glucagon at 1 mM glucose (panel C, n = 54, r = -0.27, p = 0.11) in human islets.

The mRNA expression of the SGTL2 (*SLC5A2*) and SGLT1 (*SLC5A1*) genes was analysed in RNA extracted from human islet donors (n = 131) using RNA sequencing. Expression of *SLC5A1* (mean log2CPM = 5.26) was much higher than the very low expression of *SLC5A2* (mean log2CPM = -3.19. As a comparison glucagon has a mean expression of log2CPM = 15.02 and insulin log2CPM = 10.16). As disruption of *SLC5A2* by siRNA has been shown to influence glucagon (*GCG*) secretion [14] we further explored this by co-expression analyses. Expression of *SLC5A2* did show a strong correlation with the insulin gene (n = 131, r = 0.63, q-value<0.0001), but not with the glucagon gene (Fig 4B, n = 131, r = -0.15, q-value = 0.19) or glucagon secretion at 1 mM glucose (n = 54, r = -0.27, p = 0.11). *SLC5A1* correlated with glucagon (Fig 4A, n = 131, r = 0.53, q-value<0.0001), but not with insulin (n = 131, r = -0.15, q-value = 0.11) expression.

## Discussion

The molecular genetic analyses demonstrated that the renal glycosuria in this family was due to mutations in the *SLC5A2* gene. While most of the individuals were heterozygous for a deletion or a missense mutation in the *SLC5A2* gene, the index case, a person with more severe symptoms was compound heterozygous for both mutations. Given the long follow-up of this family as part of the Botnia study [18] we could also examine whether chronic loss of glucose in the urine had any influence on the changes in the glucose tolerance or the BMI. However, we did not find any such effect, but we need to keep in mind that these data are restricted to a single although large pedigree and we can therefore not generalize this observation to all carriers of mutations in the *SLC5A2* gene.

In the majority of other reported cases of familial renal glycosuria the causal mutation has been located in the protein coding sequence of the *SLC5A2* gene [35]. In our study a novel frame shift deletion (c.300-303+2del) spanning the end of exon 3 and part of the splice site in intron 3 of the *SLC5A2* gene was found to be associated with glycosuria. All individuals carrying this novel mutation in its heterozygous form tested repeatedly positive for glucose in the urine (Fig 2A). In keeping with these findings heterozygous mutations in *SLC5A2* have previously been shown to result in mild symptoms of FRG.

The deletion causes a frame shift that is predicted to introduce a stop codon at amino acid residue 117, causing premature end of translation and resulting in a truncated peptide (S1 Fig) with reduced function, as predicted by snpEff [23]. The resulting peptide would lack the transmembrane helices that are necessary for glucose transport [36].

The index case, a female member of the pedigree (No 45) had shown more severe symptoms including postprandial hypoglycemia as well as chronic urinary and genital infections, predominantly mycotic candida infections. Because of the severe symptoms she was examined at the Helsinki University Central Hospital in the 1970’s with a kidney biopsy that showed no specific histological changes. However, she had excreted glucose already at low plasma glucose concentrations.

We therefore hypothesized that she would have been homozygous for the deletion found in the other family members. Unfortunately, we could not include her DNA in the exome sequencing efforts as she had died from a gastric tumor in 1989, and no DNA had been taken in the 70ies. We however imputed her genotype from her husband, daughter and grandchildren and the most likely segregating variant in the *SLC5A2* gene was a p.A343V mutation, which we confirmed by genotyping DNA from her gastric biopsy. The missense mutation (p.A343V) is located in a helical structure in the hydrophilic chains on the extracellular side of the membrane based on homology modeling of SGLT2 (SWISS-MODEL Repository [37,38]). This helix together with another extracellular helical structure is supposed to be important for conformational changes necessary for sodium and glucose transport [36]. This was also tested by expressing the mutation in HEK 293 cells, where it resulted in impaired glucose transport (Fig 3). It is therefore likely that the index case carried two damaging mutations in the *SLC5A2* gene resulting in an extremely low threshold for glucose reabsorption in the proximal tubule.

Recently a novel class of drugs has been introduced for the treatment of type 2 diabetes, namely SGLT2 inhibitors [16,39–41] that mimic the effect of the mutations described above. Taking advantage of the long follow-up of these family members, we also analyzed whether chronic glycosuria would have beneficial effects on changes in the glucose tolerance over time.

Whereas the level of glycemia is strongly correlated with the degree of glycosuria in a population with diabetes, this was not the case in the mutation carriers. Mutation carriers also did not show less increase in AUC_OGTT_ or HbA_1c_ over time than non-carriers of the mutation (Fig 2D) as would be expected if chronic glycosuria would protect against deterioration of glycemia and development of diabetes.

Although SGLT2 is predominantly found in the proximal tubule, it has been shown that the alpha cells of human pancreatic islets also express SGLT2 as well as the high-affinity, low-capacity transporter SGLT1 [14]. We confirmed the expression of these transporters at mRNA level in 131 islet preparations from human cadaver donors. It has been suggested that *SLC5A2* affects glucagon secretion in human islets [14], but we did not observe any correlation between *SLC5A2* and *GCG* mRNA or the glucagon secretion in the large islet sample (Fig 4B). Instead the more highly expressed *SGLT1* gene was strongly correlated with the expression of the glucagon gene (Fig 4A). However, in contrast to previous data [14], this expression was not influenced by the diabetic status of the donor. Interestingly, *SLC5A2* was positively correlated with the insulin gene, the consequence of which is unknown.

In conclusion, mutations in the SLC5A2 gene were the most likely cause of renal glycosuria in this family. Compound heterozygosity of mutations in the *SLC5A2* gene was associated with a severe form of the disease characterized by chronic urogenital candida infections and postprandial hypoglycemia. In this family we found no evidence that chronic loss of glucose in the urine would protect from deterioration of the glucose tolerance over time. This could be another example of difficulties in targeting homeostatic mechanisms in human metabolism.

## Supporting Information

S1 Fig Alignment of the wild type and mutant *SLC5A2* RNA translation to amino acids. Wild type is the native sequence while deletion is from the RNA with the deleted 4 bases from c.300-303+2del (2 deleted bases in the intronic region of the gene are not considered for simplicity). The red portion of the deletion sequence aligns perfectly with the native protein sequence, but the remaining sequence is frame shifted due to the deletion and shortly after the mutation a stop codon is introduced (here represented by |).
(PDF)

S2 Fig Genotyping using genotype specific primers showed that the index case (45) was a carrier of the *SLC5A2* p.A343V mutation. The figure shows the gel picture from a tapestation analysis where lane B1 and C1 contains PCR product from the index case, lane D1 and E1 are positive controls for the reference C-genotype, lane F1 and G1 are positive controls for the alternative T-genotype and lane H1 and A2 are negative controls for the T-allele.
(PDF)

S3 Fig The glucose tolerance at the first and last visit. A) The median AUC_OGTT_ value for all the individuals at the first and the last visit. B) The absolute AUC_OGTT_ for the non-carriers and the carriers of the *SLC5A2* c.300-303+2del mutation at the first and the last visit.
(TIF)

S4 Fig Individual family member’s OGTT-curves at the first and the last visit. (TIF)

S5 Fig BMI at first and last visit. A) The median BMI for all the individuals at the first and the last visit. B) The BMI for the non-carriers and the carriers of the *SLC5A2* c.300-303+2del mutation at the first and the last visit.
(TIF)

S6 Fig The glucose uptake in *SLC5A2* transfected cells is 10 times higher compared to untransfected cells. Panel A shows the total amount of DNA in lysate from wells used for glucose uptake assay (untransfected n = 2, transfected n = 6). Panel B show the relative glucose uptake in untransfected and wild type *SLC5A2* transfected HEK293 cells normalized to the total DNA content of the wells (untransfected n = 2, transfected n = 3). * p<0.05, *** p<0.001.
(TIF)

S1 Table The cDNA sequence used for gene synthesis of wild type and mutant *SLC5A2* to be cloned into the pCMV6-Neo vector. The constructs were transfected into HEK293 cell lines for glucose uptake studies.
(PDF)

S2 Table The oligonucleotide sequences used for the genotyping of p.A343V in the index case. The annealing temperature and the number of PCR cycles are indicated in the table.
(PDF)
