# Supplementary material for: Influence of Familial Renal Glycosuria Due to Mutations in the SLC5A2 Gene on Changes in Glucose Tolerance over Time
Source: PLoS One. 2016 Jan 6;11(1):e0146114. doi: 10.1371/journal.pone.0146114 (PMC4703216; doi:10.1371/journal.pone.0146114)
Supplement: S1 Fig — Wild type is the native sequence while deletion is from the RNA with the deleted 4 bases from c.300-303+2del (2 deleted bases in the intronic region of the gene are not considered for simplicity). The red portion of the deletion sequence aligns perfectly with the native protein sequence, but the remaining sequence is frame shifted due to the deletion and shortly after the mutation a stop codon is introduced (here represented by |). (PDF) [file pone.0146114.s001.pdf]

```
Wild type    MEEHTEAGSAPEMGAQKALIDNPADILVIAAYFLLVIGVGLWSMCRTNRGTVGGYFLAGR
             ::::::::::::::::::::::::::::::::::::::::::::::::::::::::::::
Deletion     MEEHTEAGSAPEMGAQKALIDNPADILVIAAYFLLVIGVGLWSMCRTNRGTVGGYFLAGR

Wild type    SMVWWPVGASLFASNIGSGHFVGLAGTGAASGLAVAGFEWNALFVVLLLGWLFAPV YLTA
             ::::::::::::::::::::::::::::::::::::::::
Deletion     SMVWWPVGASLFASNIGSGHFVGLAGTGAASGLAVAGFEWRSSWCCYWAGCLHPCT|QRGS

Wild type    GVITMPQYLRKRFGGRRIRLYLSVLSLFLYIFTKISVDMFSGAVFIQQALGWNIYASVIA

Deletion     SRCHSTCASASAAAASASTCLCSPFSCTSSPRSQWTCSPELYSSSRLWAGTSMPPSSRFW

Wild type    LLG ITM IYTVTG GLAALMYTDTVQTFVILGGACILMGYAFHEVGGYSGLFDKYL GAATSL

Deletion     ASP|FTR|QEGWPR|CTRTRYRPSSFWGAPASSWVTPSTRWAGIRVSSTNTWEQRLR|RCPRIQ

Wild type    TVSEDPAVGNISSFCYRPRPDSY HLLRHPVTGDLPWPALLLGLTIVSGWYWCSDQVIVQ R

Deletion     PWETSPASAIDPGPTPTTCSGTP|PGICRGPRCSSDSQSSRAGTGAATRSSCSAAWPGRA|P

Wild type    CLAGKSLTHIK A GCILCGYLKL TPMFLMVMPGMISRILYPDEVACVVPEVCRRVCGTEV G

Deletion     TSRRAASCVGT|S|RPCFSWSCQA|SAAFCTQTRWRAWCLRCAGACAARRWAAPTSPTRGSS|S

Wild type    CSNIAYPRLVVKLMPNGLRGLMLAVMLAALMSSLASIFNSSSTLFTMDIYTRLRPRAGDR

Deletion     SCPTVCADSCWRSCWPRSCPRWPPSSTAAARSSPWTSTRACGHAPATASCCWWDGSGWCS

Wild type    EL LLVGRLWVVFIVVVSVAWLPVVQAAQGGQLFDYIQAVSSYLAPPVSAVFVLALFVPRV

Deletion     SW|CRWPGFPWCRRHRAGSSSITSRQSLATWHRPCPPSSCWRSSCRALMSRAPSGDSSGAC|

Wild type    NEQGA FWGLIGGLLMGLARLIPEFSFGSGSCVQPSACPAFLCGVHYLYFAIVLFFCSGLL

Deletion     WAWHA|FPSSPSARAAVCSPRRAQLSSAACTTSTSPLCCSSALASSPSRSPCAPRPSPEST

Wild type    TLTVSLCTAPIPRKHLHRLVFSLRHSKEEREDLDADEQQG SSLPVQNGCPESAMEMNEPQ

Deletion     STAWSSVSGIARRNGRTWMLMSSKAPHSLYRMGAQRVPWR|MSPRPRHQASSASACSGFVE|

Wild type    APAPSLFRQCLLWFCGMSRGGVGSPPPLTQEEAAAAARRLED ISEDPSWARVVNLNALLM

Deletion     AEVGWAVLRPLPRRRQRQQPGGWRTSARTRAGPVWSTSMPCS|WQWPCSSGASMP

Wild type    MAVAVFLWGFYA|

Deletion
```
