# Supplementary figures and images for: Influence of Familial Renal Glycosuria Due to Mutations in the SLC5A2 Gene on Changes in Glucose Tolerance over Time
Source: PLoS One. 2016 Jan 6;11(1):e0146114. doi: 10.1371/journal.pone.0146114 (PMC4703216; doi:10.1371/journal.pone.0146114)

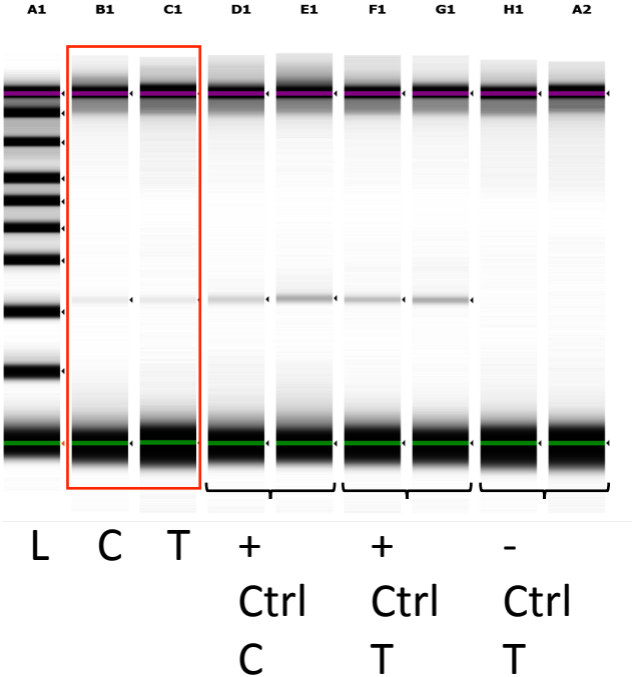

Supplement: S2 Fig — The figure shows the gel picture from a tapestation analysis where lane B1 and C1 contains PCR product from the index case, lane D1 and E1 are positive controls for the reference C-genotype, lane F1 and G1 are positive controls for the alternative T-genotype and lane H1 and A2 are negative controls for the T-allele. (PDF) [file pone.0146114.s002.pdf]

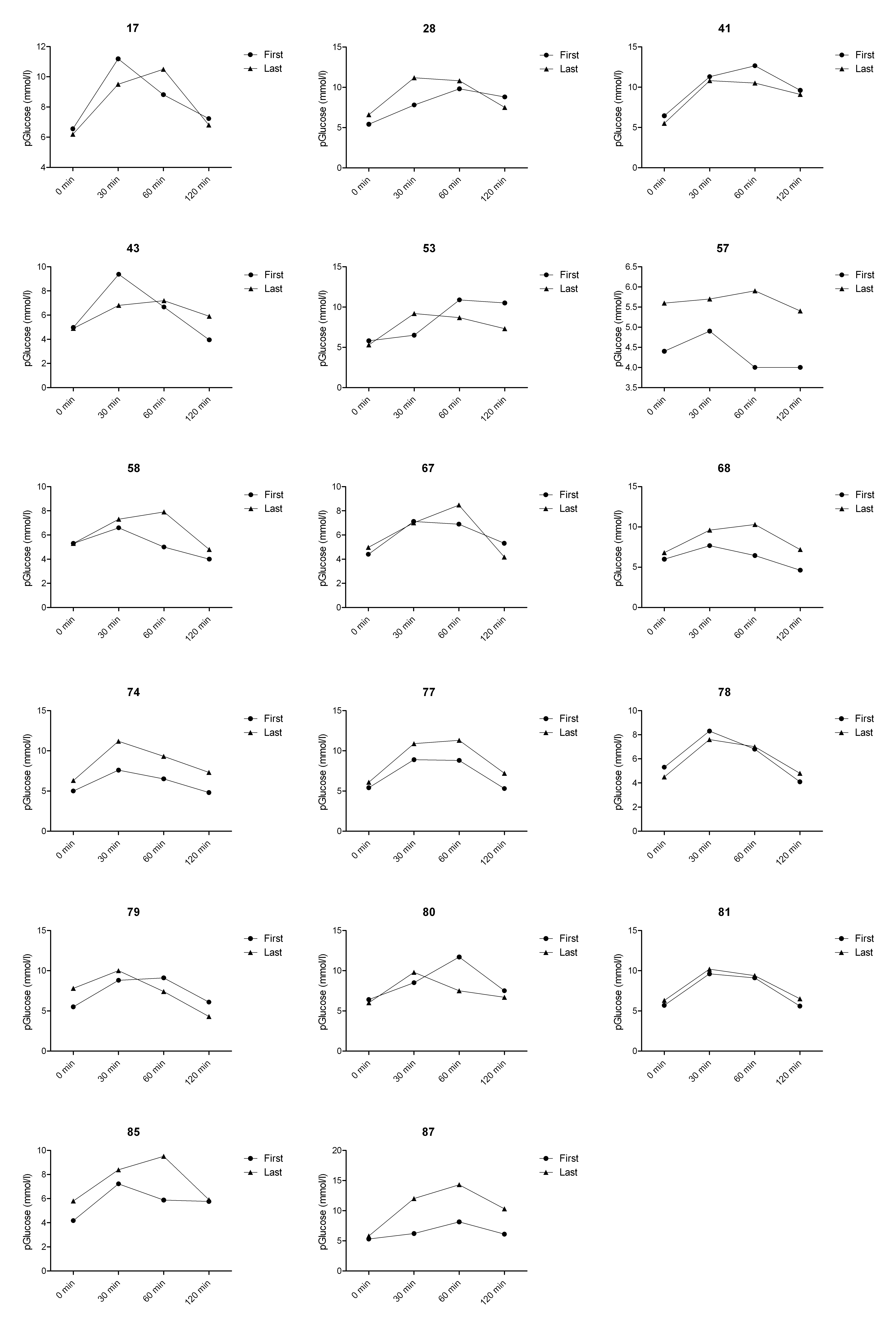

Supplement: S4 Fig — (TIF) [file pone.0146114.s004.tif]
