# Supplementary material for: Influence of Familial Renal Glycosuria Due to Mutations in the SLC5A2 Gene on Changes in Glucose Tolerance over Time
Source: PLoS One. 2016 Jan 6;11(1):e0146114. doi: 10.1371/journal.pone.0146114 (PMC4703216; doi:10.1371/journal.pone.0146114)
Supplement: S1 Table — The constructs were transfected into HEK293 cell lines for glucose uptake studies. (PDF) [file pone.0146114.s007.pdf]

cDNA sequence for *SLC5A2* gene synthesis<br><br>(Wild Type)
GGGGAATTCggggcagatcctggggagaatggaggagcacacagaggcaggctcggcaccagagatggg<br>ggcccagaaggccctgattgacaatcctgctgacatcctagtcattgctgcatatttcctgctggtcat<br>tggcgttggcttgtggtccatgtgcagaaccaacagaggcactgtgggcggctacttcctggcaggacg<br>cagcatggtgtggtggccggttggggcctctctcttcgccagcaacatcggcagtggccactttgtggg<br>cctggcagggactggcgctgcaagtggcttggctgttgctggattcgagtggaatgcgctcttcgtggt<br>gctgctactgggctggctgtttgcacccgtgtacctgacagcgggggtcatcacgatgccacagtacct<br>gcgcaagcgcttcggcggccgccgcatccgcctctacctgtctgtgctctcccttttcctgtacatctt<br>caccaagatctcagtggacatgttctccggagctgtattcatccagcaggctctgggctggaacatcta<br>tgcctccgtcatcgcgcttctgggcatcaccatgatttacacggtgacaggagggctggccgcgctgat<br>gtacacggacacggtacagaccttcgtcattctgggggcgcctgcatcctcatgggttacgccttcca<br>cgaggtgggcgggtattcgggtctcttcgacaaatacctgggagcagcgacttcgctgacggtgtccga<br>ggatccagccgtgggaaacatctccagcttctgctatcgaccccggcccgactcctaccacctgctccg<br>gcaccccgtgaccggggatctgccgtggcccgcgctgctcctcggactcacaatcgtctcggctggta<br>ctggtgcagcgaccaggtcatcgtgcagcgctgcctggccgggaagagcctgacccacatcaaggcggg<br>ctgcatcctgtgtgggtacctgaagctgacgcccatgtttctcatggtcatgccaggcatgatcagccg<br>cattctgtacccagacgaggtggcgtgcgtggtgcctgaggtgtgcaggcgcgtgtgcggcacggaggt<br>gggctgctccaacatcgcctacccgcggctcgtcgtgaagctcatgcccaacggtctgcgcggactcat<br>gctggcggtcatgctggccgcgctcatgtcctcgctggcctccatcttcaacagcagcagcacgctctt<br>caccatggacatctacacgcgcctgcggccacgcgccggcgaccgcgagctgctgctggtgggacggct<br>ctgggtggtgttcatcgtggtagtgtcggtggcctggcttcccgtggtgcaggcggcacagggcgggca<br>gctcttcgattacatccaggcagtctctagctacctggcaccgcccgtgtccgccgtcttcgtgctggc<br>gctcttcgtgccgcgcgttaatgagcagggcgccttctggggactcatcgggggcctgctgatgggcct<br>ggcacgcctgattcccgagttctccttcggctcgggcagctgtgtgcagccctcggcgtgcccagcttt<br>cctctgcggcgtgcactacctctacttcgccattgtgctgttcttctgctctggcctcctcaccctcac<br>ggtctccctgtgcaccgcgcccatccccagaaagcacctccaccgcctggtcttcagtctccggcatag<br>caaggaggaacgggaggacctggatgctgatgagcagcaaggctcctcactccctgtacagaatgggtg<br>cccagagagtgccatggagatgaatgagccccaggccccggcaccaagcctcttccgccagtgcctgct<br>ctggttttgtggaatgagcagaggtggggtgggcagtcctccgccccttacccaggaggaggcagcggc<br>agcagccaggcggctggaggacatcagcgaggacccgagctgggcccgtgtggtcaacctcaatgccct<br>gctcatgatggcagtggccgtgttcctctggggcttctatgcctaagaccaactgcgttggacaccata<br>agccacagcctcacaggaagtggggtgaggagcctgcggtgctccccagaaaggggaaggggcagtg<br>gggtgagaaggtcctggctccccttctcccggccttcctctgcctggggcccactgcatctgattggca<br>gtcacttcccatgagggcctggcccacccgctgcagttgccctaaggaaaaataaagctgcctttcccc<br>tgtccaaaaaaTCTAGAGGGGG
cDNA sequence for *SLC5A2* cloning<br><br>(Mutant p.A343V)
GGGGAATTCggggcagatcctggggagaatggaggagcacacagaggcaggctcggcaccagagatggg<br>ggcccagaaggccctgattgacaatcctgctgacatcctagtcattgctgcatatttcctgctggtcat<br>tggcgttggcttgtggtccatgtgcagaaccaacagaggcactgtgggcggctacttcctggcaggacg<br>cagcatggtgtggtggccggttggggcctctctcttcgccagcaacatcggcagtggccactttgtggg<br>cctggcagggactggcgctgcaagtggcttggctgttgctggattcgagtggaatgcgctcttcgtggt<br>gctgctactgggctggctgtttgcacccgtgtacctgacagcgggggtcatcacgatgccacagtacct<br>gcgcaagcgcttcggcggccgccgcatccgcctctacctgtctgtgctctcccttttcctgtacatctt<br>caccaagatctcagtggacatgttctccggagctgtattcatccagcaggctctgggctggaacatcta<br>tgcctccgtcatcgcgcttctgggcatcaccatgatttacacggtgacaggagggctggccgcgctgat<br>gtacacggacacggtacagaccttcgtcattctgggggcgcctgcatcctcatgggttacgccttcca

cgaggtgggcgggtattcgggtctcttcgacaaatacctgggagcagcgacttcgctgacggtgtccga
ggatccagccgtgggaaacatctccagcttctgctatcgaccccggcccgactcctaccacctgctccg
gcaccccgtgaccggggatctgccgtggcccgcgctgctcctcggactcacaatcgtctcgggctggta
ctggtgcagcgaccaggtcatcgtgcagcgctgcctggccgggaagagcctgacccacatcaaggcggg
ctgcatcctgtgtgggtacctgaagctgacgcccatgtttctcatggtcatgccaggcatgatcagccg
cattctgtacccagacgaggtggtgtgcgtggtgcctgaggtgtgcaggcgcgtgtgcggcacggaggt
gggctgctccaacatcgcctacccgcggctcgtcgtgaagctcatgcccaacggtctgcgcggactcat
gctggcggtcatgctggccgcgctcatgtcctcgctggcctccatcttcaacagcagcagcacgctctt
caccatggacatctacacgcgcctgcggccacgcgccggcgaccgcgagctgctgctggtgggacggct
ctgggtggtgttcatcgtggtagtgtcggtggcctggcttcccgtggtgcaggcggcacagggcgggca
gctcttcgattacatccaggcagtctctagctacctggcaccgcccgtgtccgccgtcttcgtgctggc
gctcttcgtgccgcgcgttaatgagcagggcgccttctggggactcatcgggggcctgctgatgggcct
ggcacgcctgattcccgagttctccttcggctcgggcagctgtgtgcagccctcggcgtgcccagcttt
cctctgcggcgtgcactacctctacttcgccattgtgctgttcttctgctctggcctcctcaccctcac
ggtctccctgtgcaccgcgcccatccccagaaagcacctccaccgcctggtcttcagtctccggcatag
caaggaggaacgggaggacctggatgctgatgagcagcaaggctcctcactccctgtacagaatgggtg
cccagagagtgccatggagatgaatgagccccaggccccggcaccaagcctcttccgccagtgcctgct
ctggttttgtggaatgagcagaggtggggtgggcagtcctccgccccttacccaggaggaggcagcggc
agcagccaggcggctggaggacatcagcgaggacccgagctgggcccgtgtggtcaacctcaatgccct
gctcatgatggcagtggccgtgttcctctggggcttctatgcctaagaccaactgcgttggacaccata
agccacagcctcacaggaagtggggtgaggagcctgcggtgctccccagaaaggggaagggcagtg
gggtgagaaggtcctggctccccttctcccggccttcctctgcctggggcccactgcatctgattggca
gtcacttcccatgagggcctggcccacccgctgcagttgccctaaggaaaaataaagctgcctttcccc
tgtccaaaaaaTCTAGAGGGGG
