# Supplementary material for: Influence of Familial Renal Glycosuria Due to Mutations in the SLC5A2 Gene on Changes in Glucose Tolerance over Time
Source: PLoS One. 2016 Jan 6;11(1):e0146114. doi: 10.1371/journal.pone.0146114 (PMC4703216; doi:10.1371/journal.pone.0146114)
Supplement: S2 Table — The annealing temperature and the number of PCR cycles are indicated in the table. (PDF) [file pone.0146114.s008.pdf]

	Oligo sequence	Annealing temperature	# PCR cycles
Forward C-allele	5’ TCTGGCAGACGAGGTGGC 3’	67°C	40
Forward T-allele	5’ TCTGGCAGACGAGGTGGT 3’		
Reverse primer (used in both reactions)	5’TTACCGTTGGGCATGAGCTT 3’		
